# Misoprostol, Magnesium Sulphate and Anti-shock garment: A knowledge, availability and utilization study at the Primary Health Care Level in Western Nigeria

**DOI:** 10.1371/journal.pone.0213491

**Published:** 2019-03-21

**Authors:** Adeola O. Duduyemi, Ifeoma P. Okafor, Ezekiel S. Oridota

**Affiliations:** Department of Community Health and Primary Care, College of Medicine, University of Lagos, Lagos, Nigeria; University of Rwanda, RWANDA

## Abstract

**Introduction:**

Nigeria has one of the highest maternal mortality ratios in the world. The nurses and midwives being the first point of contact play a central role in addressing these problems. This study was conducted to assess the knowledge and utilization of the technologies (misoprostol, anti-shock garment and magnesium sulphate) in the reduction of maternal mortality amongst the Primary Health Care (PHC) nurses and midwives in Lagos State, Nigeria. In addition, the availability of the technologies in the flagship Primary Health Centres (PHCs) was assessed.

**Methods:**

This was a cross-sectional study among all the nurses and midwives at the flagship PHCs in Lagos state and a total of 230 were eventually studied. Data was collected using a self-administered, structured questionnaire and a checklist. Descriptive and inferential statistics were applied. Level of significance was set at 5% (p<0.05).

**Results:**

All the respondents were aware of the technologies but most (73.9%) had poor knowledge of them. Majority (74.8%) of the respondents had good knowledge of maternal mortality and its major causes. Most, 81.3% of the respondents have administered misoprostol, 37.0% magnesium sulphate while 52.2% have administered anti shock garment. Out of the 57 flagship PHCs, 27 (47.4%) had magnesium sulphate, 42 (73.7%) had misoprostol and 52 (91.2%) had anti-shock garments in their facilities. Respondents who were double qualified (nurse/midwife) had significantly better knowledge of maternal mortality and its major causes (p = 0.009) than the other cadres. Longer years of experience (p = 0.019), training in the use of misoprostol (p = 0.020) and training in the use of magnesium sulphate (p = 0.001) significantly improved knowledge of the technologies.

**Conclusion:**

Respondents had good knowledge of maternal mortality and its major causes and poor knowledge of the technologies for maternal mortality reduction, despite the trainings attended. Of the three technologies considered, misoprostol was the most commonly used. Periodic refresher courses for the training and retraining of PHC nurses and midwives on the technologies for maternal mortality reduction is recommended.

## Introduction

Nigeria has one of the highest ratio of maternal mortality in the world, with a maternal mortality ratio of about 576 per 100,000 live births. A woman’s chance of dying from pregnancy and childbirth in Nigeria is 1 in 13 [1]. Nigeria loses about 145 women of childbearing age every single day, this makes the country the second largest contributor to maternal mortality rate in the world [2]. Comparatively, the global average risk is 1 in 180, and the risk of women of childbearing age dying in developed countries is 1 in 3,800. The leading causes of maternal mortality in Nigeria mirror those of similar developing countries around the world. An estimated 23% of maternal deaths are due to postpartum hemorrhage; 17% due to sepsis; and another 44% of deaths are due to an equal burden of eclampsia, unsafe abortion, obstructed labor, and anaemia [3]. A woman’s risk of death from one of these causes decreases significantly if she gives birth in the presence of a skilled attendant.

In 2003, a group of maternal health experts met in Bellagio, Italy to deliberate on technologies that would address the five leading causes of maternal mortality which are post-partum haemorrhage (PPH), eclampsia, obstructed labour, puerperal sepsis and unsafe abortion [4]. Five technologies were identified as priorities during the Bellagio workshop, three related to PPH prevention (active management of third stage of labour [AMTSL], misoprostol, and oxytocin in the Uniject device) and two to treatment of serious haemorrhage (antishock garment and balloon tamponade). Technologies such as Magnesium sulphate for prevention and treatment of eclampsia, use of antihypertensive drugs in women with mild-to-moderate hypertension to prevent pre-eclampsia and use of nutritional supplements, and antiplatelets to prevent preeclampsia and eclampsia were also discussed [4]. It will be difficult to achieve fifth millenium development goal (MDG5) which is aimed at improving maternal health without these technologies. These technologies (anti-shock garment, misoprostol and magnesium sulphate) have been instituted in Lagos State health facilities, particularly all the flagship health centers, to help reduce maternal mortality in the state [5]. The high maternal mortality has remained a huge challenge hence the study of the 57 flagship PHCs where these technologies are meant to be available. Since nurses and midwives take deliveries and manage complications of pregnancy and childbirth in the PHCs, an increased focus on improving their skills and making availale the technologies in these basic health care facilities would bring about improvements in maternal outcomes necessary to meet MDG five.

On the basis of the available evidence, The World Health Organization (WHO) has recommended Magnesium Sulphate (MgSO4) as the most effective, safe, and low-cost drug for the treatment of severe pre-eclampsia and eclampsia [6]. There are indeed several reports of its successful introduction in several countries including Nigeria and its effectiveness and safety for mother and baby [7,8].

Misoprostol has been widely recommended to prevent postpartum haemorrhage when other methods are not available [9]. Administration of this drug on a wide scale at the community level to prevent and treat postpartum haemorrhage is of major public health importance. In more recent studies misoprostol has proved better than placebo, in terms of measured blood loss, for both the prevention [10,11] and treatment [12] of postpartum haemorrhage.

The non-pneumatic anti-shock garment (NASG) is a low-technology pressure device which decreases blood loss, restores vital signs, and has the potential to improve adverse outcomes by helping women survive delays in receiving adequate emergency obstetric care. With brief training, even individuals without medical backgrounds can apply this first-aid device [13]. In many low-resource settings where there are delays in transport to referral facilities in order to obtain lifesaving treatments, the NASG device can be used to prevent maternal deaths due to obstetric haemorrhage. Improving the knowledge and practice of the technologies among the nurses and midwives is a promising strategy to effectively reduce maternal mortality.

A study in a large teaching hospital in England to promote evidence-based practice among nurses examined the extent to which nurses utilised different sources of knowledge to inform their practice and findings showed that nurses relied most heavily on experiential knowledge gained through their interactions with nursing colleagues, medical staff and patients to inform their practice. Whereas nurses were relatively well skilled at accessing and reviewing research evidence, they were less confident about their ability to change practice [14].

A cross-sectional study of 131 PHCs and 148 higher referral facilities (74 public and 74 private) in eight districts of the region was conducted in India in 2014. For the test case on PPH, only 37.7% of the providers would assess for uterine tone, and 40% correctly defined PPH. In this study, magnesium sulphate, the drug of choice to control convulsions in eclampsia was available in 18% of PHCs, 48% of higher public facilities and 70% of private facilities. In response to the test case on eclampsia, 54.1% and 65.1% of providers would administer anti-hypertensives and magnesium sulphate, respectively; 24% would administer oxygen and only 18% would monitor for magnesium sulphate toxicity [15].

In a descriptive, non-experimental survey conducted in the two government tertiary hospitals in Yenagoa Local Government Area of Bayelsa State Nigeria, amongst 80 midwives, a majority (85%) had a high level knowledge of strategies used in the prevention and control of PPH while 60 (75%) of respondents had managed PPH. Amongst the 80 midwives, 78(97.5%) accepted that uterotonics are used in the management of PPH, while 63 (78.8%) noted that oxytocin was the one commonly used, followed by 16 (20%) respondents who reported that Ergometrine was commonly used [16].

A study was done among 102 Specialist obstetricians and gynaecologists during the International conference of Society of Gynaecology and Obstetrics of Nigeria (SOGON) in Abuja, Nigeria in November 2006 using self-administered structured questionnnaires. The majority (64.7%) of respondents were between 25 and 45 years. Majority (93%) of respondents had used misoprostol in their clinical practice. Out of these, 44% had used it for the management of early pregnancy failure and 43% for postpartum haemorrhage. Most (89%) of the respondents were satisfied with the efficacy and tolerability of misoporstol while 9% expressed partial satisfaction. Misoprostol was widely available in the environment of 82% of the respondents, whilst 14% admitted that it was either seldom or not available. Despite the popularity of the drug in Nigeria, only about 53% of respondents were aware that misoprostol has been registered for use in the management of post-partum haemorrhage [17].

The study aimed to assess the knowledge and the use of antishock garment, misoprostol and magnesium sulphate in the reduction of maternal mortality by nurses and midwives in the flagship PHCs in Lagos State, Nigeria. In addition, the availability of these technologies in the PHCs will be ascertained. Existing gaps may then be identified and appropriate interventions instituted to further reduce the high maternal mortality and morbidity experienced in the country.

## Materials and methods

### Study area

Lagos state is the smallest state in Nigeria with an area of 356,861 hectares of which 75,755 hectares are wetlands. The state is located in the southwestern part of Nigeria. It was created on May 27, 1967 [18,19].

Lagos state has about four million women of child bearing age which is 22.9% of the entire population [20]. There are two hundred and seventy seven (277) PHCs under the supervision of Lagos State Primary Health Care Board. Two of the PHCs which are not flagship centers were visited for pre-testing and the questionnaire was pretested on 10 nurses and midwives. After pretesting, adjustments were made on the questionnaires based on findings before the actual study was carried out.

There are 57 flagship PHCs in the state with an estimate of 1,296 deliveries per month and all these deliveries are Spontaneous Vaginal Deliveries (SVD) [21]. These 57 PHCs are called flagship because they have been upgraded and equipped to provide basic emergency obstetric care and also to run for 24 hours. Some health workers including midwives were trained recently prior to the study on the use of these technologies [5]. At the time of the study, there were 677 nurses/ midwives employed in PHCs in Lagos state and not less than 245 of them were in the flagship PHCs [21, 22].

### Sample size and sampling procedure

This was a cross-sectional study among all the nurses and midwives at the flagship PHCs in Lagos state i.e. total population. A total of 230 out of 245 nurses and midwives were eventually studied.

### Data collection and quality checking

The data was collected over a period of six weeks. The study involved quantitative data collection tools by the use of self-administered, structured questionnaire and a checklist on the availability of the technologies. The questionnaires were administered to the respondents at work. Some respondents were not met at their duty posts during the first visit because they usually run shifts and appointments were rescheduled to when they were on duty. A checklist was used to assess the availability of the technologies in the facilities. The principal researcher interviewed the nurse/midwife in charge of the PHCs for this purpose. The results were analyzed using SPSS software. Pearsons Chi Square was used to assess statistical significance between selected variables and the level of significance was set at 5% (p< 0.05).

### Ethical consideration

Ethical approval was obtained from Health Research Ethics Committee of the Lagos University Teaching Hospital (LUTH). Permission to conduct the study was obtained from the Lagos State Primary Health Care Board. The participants were assured of anonymity and confidentiality Throughout the study, there were no risks involved in the study.

## Results

Following the calculated minimum sample size of 228, a total of 245 questionnaires were distributed among the respondents. Two hundred and thirty seven questionnaires were retrieved (96.7% response rate) out of which 230 were valid for analysis.

Over two-third of the respondents in this study are nurse/midwife (69%), midwife (14%), CHO/nurse (9%), and nurse (8%). Hence, nearly all of them are females (94%). Their ages were almost evenly distributed (about 30%) between age groups 30–39 years, 40–49 years, 50 years and above, while only 11% of them were less than 30 years. The mean age of the entire groups is 41.90 ±9.43 years. The mean years of experience of the healthcare providers is 16.23± 9.54 years. Table 1

**Table 1:** Sociodemographic characteristics of respondents.

Variable (n = 230)	Frequency	Percentage (%)
**Qualification**		
Nurse	19	8.3
Midwife	33	14.3
Community health officer/midwife	20	8.7
Nurse/midwife	158	68.7
**Gender**		
Male	14	6.1
Female	216	93.9
**Age**		
20–29 years	26	11.3
30–39 years	65	28.3
40–49 years	71	30.9
50 years and above	68	29.6
Mean = 41.90 ±9.43 years		
**Years of experience as a healthcare provider**		
10 years and below	99	44.6
11–20 years	44	19.8
21–30 years	66	29.7
31–40 years	13	5.9
Mean = 16.23± 9.54		

### Knowledge of major causes of maternal mortality

The causes of maternal mortality in developing countries including Nigeria reported by the respondents included postpartum haemorrhage (192; 83.5%), unsafe abortion (168; 73.0%), pre-eclampsia and eclampsia (161; 70.0%). Most 208 (90.4%) of the respondents reported that misoprostol can be used to manage PPH, 116 (50.4%); induction of labour and 55 (23.9%); abortion. Few 13 (5.7%) chose 800ug sublingual as the dosage of misoprostol for the management of PPH which is the correct dose and most 87 (94.3) chose the wrong options. About three-quarter (210, 91.3%) of the respondents know that anti-shock garment is used as first aid device to reverse hypovolaemic shock while 20(9.7%) gave the incorrect answers. More than three-quarter (182, 79.1%) of the respondents reported that patients need anti-shock garment when blood loss is greater than 500ml while 48 (20.9%) gave the incorrect answers. More than one third 92 (40.0%) of the respondents reported the correct dose of IM magnesium sulphate [6–8] and the remainder, 138 (60.0%) reported the wrong doses. Only 64 (27.8%) of the respondents knew the correct dose of IV regimen of Magnesium Sulphate. Table 2

**Table 2:** Knowledge of major causes of maternal mortality in developing countires and the technologies for its reduction.

Variable(n = 230)	Frequency	Percentage (%)
Major causes of maternal mortality*		
Postpartum haemorrhage	192	83.5
Unsafe abortion	168	73.0
Pre-eclampsia and eclampsia	161	70.0
Obstructed labour	138	60.0
Sepsis	130	56.5
Others	15	6.5
**Uses of misoprostol** ^*^		
Management of PPH	208	90.4
Management of induction of labour	116	50.4
Management of abortion	55	23.9
**Required dose of misoprostol in the management of PPH**		
Correct	13	5.7
Incorrect	87	94.3
**Definition of anti-shock garment**		
Correct	210	91.3
Incorrect	20	9.7
**Indication for use of anti-shock garment**		
Correct	182	79.1
Incorrect	48	20.9
**The required dose of IM magnesium sulphate**		
Correct	92	40.0
Incorrect	138	60.0
**The required dose of IV magnesium sulphate**		
Correct	64	27.8
Incorrect	166	72.2

* multiple responses

Respondents who were previously exposed to training on the use of misoprostol had significantly better knowledge of the technologies than those who were not trained (p = 0.020). Respondents who have been trained on the used of magnesium sulphate had beeter knowlege of the technologies than those who are not trained (p = 0.001). Previous training on NASG had no statistically significant effect on knowledge (p = 0.842) Table 3

**Table 3:** Association between training on the use of the technologies (Misoprostol, MgSO_4_ and NASG) and knowledge of the technologies for maternal mortality reduction.

	N	Knowledge of the technologies for maternal mortality reduction				χ^2^	df	p-value
		Poor Knowledge		Good knowledge				
		n	%	n	%			
**Training on the use of misoprostol**						5.56	1	0.020
Yes	178	125	70.2	53	29.8			
No	52	45	86.5	7	13.5			
**Training on the use of Magnesium sulphate**						13.48	1	0.001
Yes	151	100	66.2	51	33.8			
No	79	70	88.6	9	11.4			
**Training on the use of Anti-shock garment**						0.04	1	0.842
Yes	186	138	74.2	48	25.8			
No	44	32	72.7	12	27.3			
Total	230	170	73.9	60	26.1			

Though older respondents had better knowledge of maternal mortality and its major causes than others, this difference was not statistically significant (p = 0.062). Respondents that were double qualified (nurse/midwife) had significantly better knowledge of maternal mortality and its major causes than other cadres (p = 0.009). Respondents with 21–40 years of experience had better knowledge of maternal mortality and its major causes than the less experienced respondents but not with a significant difference (p = 0.260) Table 4

**Table 4:** Association between respondents’ age, qualification and work experience and knowledge of maternal mortality and its major causes.

	N	Knowledge of maternal mortality and its major causes				χ^2^	df	p-value
		Poor Knowledge		Good knowledge				
		n	%	n	%			
**Age**						7.431	3	0.062
20–29	26	12	46.2	14	53.9			
30–39	65	15	23.1	50	76.9			
40–49	71	14	19.7	57	80.2			
≥50	68	17	25.0	51	75.0			
**Designation**						11.549	3	0.009
Nurse	19	8	42.1	11	57.9			
Midwife	33	14	42.4	19	57.6			
CHO/Midwife	20	6	30.0	14	70.0			
Nurse/midwife	158	30	19.0	128	81.0			
**Years of experience**						2.70	2	0.260
≤10	99	27	27.3	72	72.7			
11–20	52	16	30.8	36	69.2			
21–40	79	15	19.0	64	81.0			
**Total**	230	58	25.2	172	74.8			

Most (190 (82.6%) of the respondents reported that they have managed a patient with PPH. Majority; (187; 81.3%) of them have administered misoprostol and 43 (18.7%) have never administered misoprostol. Of the respondents that have used misoprostol; 64 (34.2%)reported that they rarely use it; 88 (47.1%) reported that they use it sometimes, 25 (13.4%) use it often and 10 (5.3%) reported that they they use misoprostol always. A total of 85 (37.0%) of the respondents have administered magnesium sulphate to a patient. Among the respondents who have administered magnesium sulphate 79 (92.9%) of them administer it rarely, while 6 (7.1%) of them administer it sometimes. A little over half (120, 52.2%) of the respondents have administered the anti-shock garment to a patient. Among the respondents who have administered the anti-shock garment, 106 (88.3%) of them rarely administered it to a patient while 14 (11.7%) of them administered it sometimes. Table 5

**Table 5:** Utilization of the technologies among respondents.

Variable (n = 230)	Frequency	Percentage (%)
**Has managed patients with PPH**		
Yes	190	82.6
No	40	17.4
**Has administered misoprostol**		
Yes	187	81.3
No	43	18.7
**n = 187** **Frequency of use of misoprostol**		
Rarely	64	34.2
Sometimes	88	47.1
Often	25	13.4
Always	10	5.3
**Has administered magnesium sulphate**		
Yes	85	37.0
No	145	63.0
**n = 85**		
**Frequency of use of magnesium sulphate**		
Rarely	79	92.9
Sometimes	6	7.1
**Has administered anti-shock garment**		
Yes	120	52.2
No	110	47.8
**n = 187**		
**Frequency of use of anti-shock garment**		
Rarely	106	88.3
Sometimes	14	11.7

More of the respondents (90.0%) who had good knowledge administered misoprostol i.e. there was a statistically significant association between knowledge of the technologies for maternal mortality reduction and administration of misoprostol to patients (p = 0.044). There was no statistically significant association between the knowledge of the technologies for maternal mortality reduction and administration of magnesium sulphate to patients(p = 0.957). Also, there was no statistically significant association between the knowledge of the technologies for maternal mortality reduction and administration of anti-shock garment (p = 0.834). Table 6

**Table 6:** Factors associated with knowledge of the technologies for maternal mortality reduction and the utilization of the technologies (Misoprostol, MgSO_4_ and NASG).

	N	Knowledge of the technologies for maternal mortality reduction				χ^2^	Df	p-value
		Poor Knowledge		Good knowledge				
		n	%	n	%			
**Has administered misoprostol**						4.04	1	0.044
Yes	187	133	71.12	54	28.88			
No	43	37	86.05	6	13.95			
**Has administered magnesium sulphate**						0.003	1	0.957
Yes	85	63	74.12	22	25.88			
No	145	107	73.79	38	26.21			
**Has administered anti-shock garment**						0.04	1	0.834
Yes	120	88	73.33	32	26.67			
No	110	82	74.55	28	25.45			
	230	170	73.91	60	26.09			

Out of the 57 flagship PHCs, 27 (47.4%) had magnesium sulphate in their facilities, 42 (73.7%) had misoprotol in their facilities, 52 (91.2%) had anti-shock garments in their facilities. Table 7

**Table 7:** Availability of the technologies at the flagship PHCs in Lagos state.

Variable (n = 57)	Frequency	Percentage (%)
Magnesium sulphate available	27	47.4
Misoprotol available	42	73.7
Anti-shock garments available	52	91.2

## Discussion

This study has been able to assess the knowledge and utilization of the technologies for the reduction of maternal mortality among nurses and midwives in the flagship PHCs in Lagos State.

**Fig 1:**
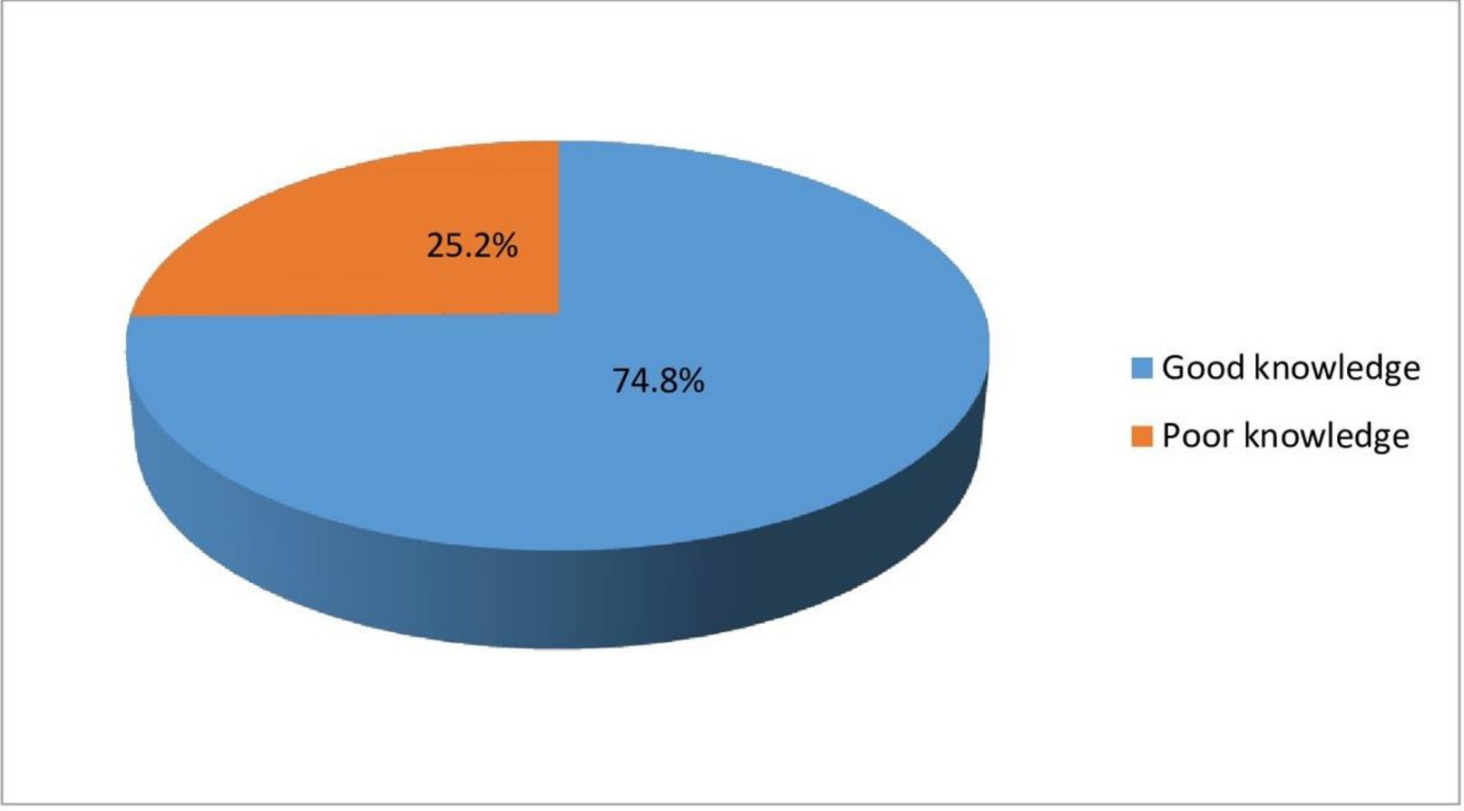
Overall knowledge of maternal mortality and its major causes among respondents. Amongst the nurses and midwives, 58 (25.2%) had poor knowledge of maternal mortality and its major causes and 172 (74.8%) of them had good knowledge (Fig 1).

**Fig 2:**
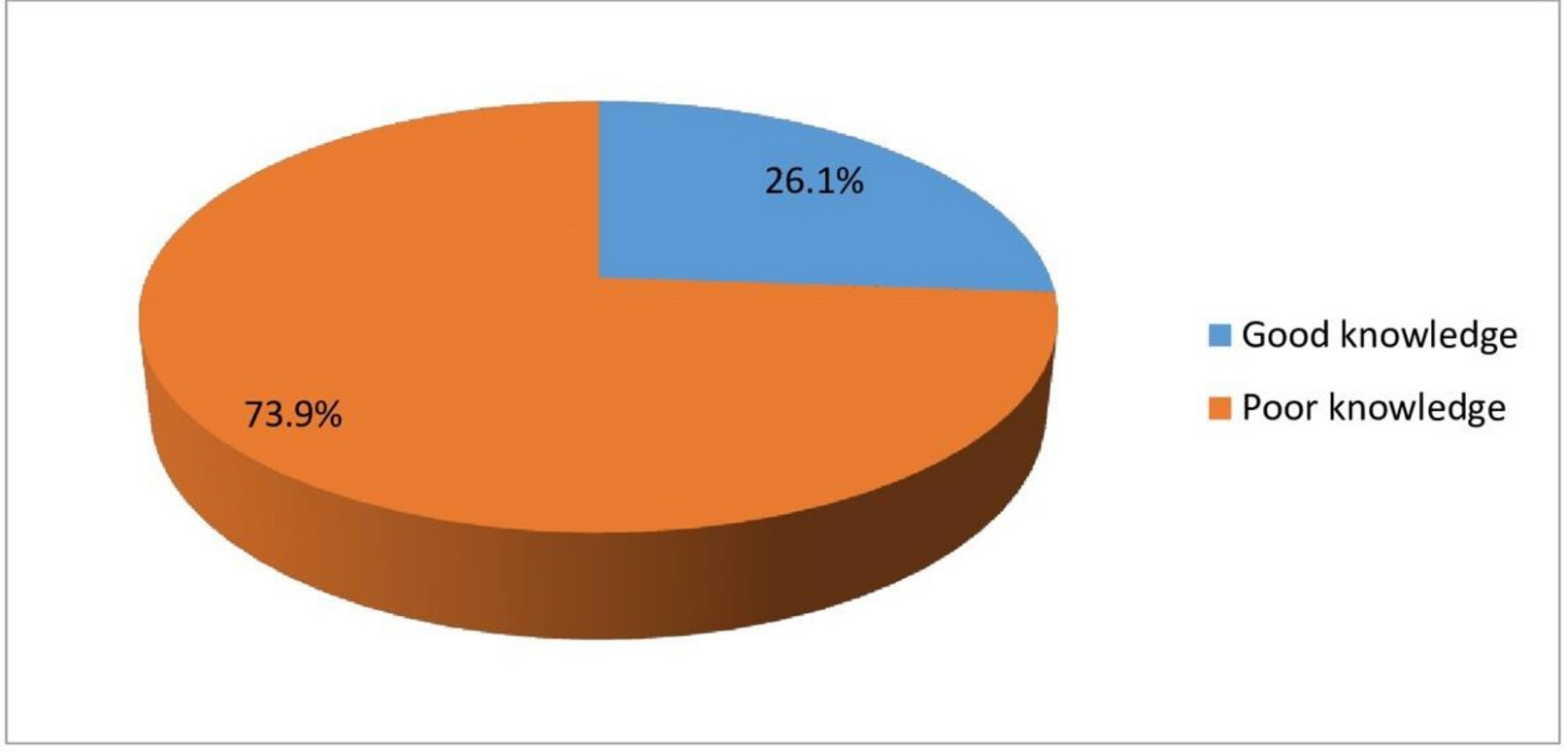
Overall knowledge of the new technologies used in the reduction of maternal mortality among respondents. Most (170;73.9%) of the nurses and midwives had poor knowledge of new technologies of maternal mortality reduction while 60(26.1%) of them had good knowledge (Fig 2).

All the respondents were aware of the the technologies for maternal mortality reduction and majority of them have been trained in all the technologies. Overall, most of the respondents (172; 74.8%) had good knowledge of maternal mortality and its major causes “Fig 1” but most of them (170;73.9%) had poor knowledge of the technologies of maternal mortality reduction “Fig 2”.

The World Health Organization endorses the important role of misoprostol in reproductive health by including it in the WHO Model List for Essential Medicines [23]. Management of PPH due to uterine atony if oxytocin has failed or is not available is 800 μg, once taken via sublingual route [24].

The study showed that 71 (30.9%) respondents were between 40–49 years and the mean age of the respondents was determined to be 41.90 ±9.43 standard deviation.

Findings from this study revealed that most of the respondents would assess for vital signs and about half will access for uterine tone and haemoglobin level in a woman with PPH. Assessing for the aforementioned parameters in a woman with PPH will help to determine the level of PPH and thus determine the intervention to give or treatment to prescribe. Similar to this study, in the Indian study mentioned earlier, for the test case on PPH, only 37.7% of the providers would assess for uterine tone [15].

In this study, the greatest risk factor for postpartum haemorrhage, as identified by most of the respondents was mismanagement of third stage of labour. This implied that if the third stage of labour was properly managed by the respondents, the incidence of PPH will greatly reduce thus reducing maternal mortality in Lagos State. Similarly, in the Bayelsan study mentioned earlier, majority of respondents indicated that improper and mismanaged third stage of labour contributes to PPH [16].

Findings from this study revealed that the most common uteronic used in the management of PPH was misoprostol and most of the respondents reported that misoprostol can be used in the management of PPH but only 5.7% correctly stated the appropriate dose of misoprostol (800 ug sublingual). This may be because misoprostol is readily available in PHCs and they rarely give IV drugs. In PHCs nurses and midwives often take deliveries and this may be why most of the respondents were not aware of the correct dose because they learnt on the job from senior colleagues and might not have checked out literature to be sure they were doing the right thing as long as what they know is working for the patients. In a similar study in Bayelsa among midwives, almost all knew that uterotonics were used in the management of PPH and the uterotonic they reported using most was oxytocin in contrast to misoprostol used most by respondents in this study [16]. This contrast was likely due to the fact that the study was conducted in tertiary hospitals where they are more likely to give IV drugs as compared to this study which was done in PHCs.

Findings from this study showed that about three-quarter (175, 76.1%) of the nurses and midwives know that anti-shock garment is used as first aid device to reverse hypovolaemic shock. More than three-quarter (182, 79.1%) of the nurses and midwives reported that patients need anti-shock garment when blood loss is greater than 500ml.

Similarly, in the Bayelsa state study mentioned earlier, majority of the 80 respondents 59(73.8%) have heard of anti-shock garment but only 42(52.5%) know that it is used in the management of PPH [16].

Respondents that were double qualified (nurse/midwife) had significantly better knowledge of maternal mortality and its major causes than other cadres (p = 0.009). It was observed that the proportion of health care providers with good knowledge was highest (81.0%) among respondents who areare double qualified i.e nurses/midwives. This implies that health care providers who are double qualified as nurses and midwives have a better knowledge of maternal mortality and its major causes than others.

Other variables such as the age of the respondents, the years of experience as a health care provider showed no statistical significance i.e did not determine the respondents’ knowledge of maternal mortality and its major causes.

The association between years of experience of the healthcare provider and their knowledge of the technologies was found to be significant (p = 0.019). The proportion of health care providers with good knowledge of the technologies for maternal mortality reduction was highest among those with 21–40 of years experience as health care providers.

This study revealed that there was no significant association between the age, the qualification of healthcare providers and their knowledge of the technologies for maternal mortality reduction. Similarly, in the Bayelsa state study mentioned earlier, there was no significant association between the professional qualification (p = 0.349), rank (p = 0 .088) of midwives and their level of knowledge of strategies used in the prevention and management of PPH with p>0.05 [16].

About half of the respondents had their source of knowledge for the technologies as on-the-job training. This finding is consistent with findings in a study carried out in a large teaching hospital in England to promote evidence-based practice among nurses, it examined the extent to which nurses utilized different sources of knowledge to inform their practice and findings showed that nurses relied most heavily on experiential knowledge gained through their interactions with nursing colleagues, medical staff and patients to inform their practice. Whereas nurses were relatively well skilled at accessing and reviewing research evidence, they were less confident about their ability to change practice [14]. This finding is also similar to another study done in Canada on profiling Canadian Nurses' Preferred Knowledge Sources for Clinical Practice which revealed that across all units, nurses preferred to use knowledge gained through personal experience and interactions with co-workers and with individual patients rather than journal articles or textbooks [25].

The knowledge of the technologies for maternal mortality reduction significantly influenced the respondents’ administration of misoprostol (p = 0.044). This implies that most of the respondents that have good knowledge of the technologies make use of misoprostol. Therefore if the healthcare providers are well trained they will make use of the technologies and this will further reduce maternal deaths in the state.

This study revealed that there was no significant association between the administration of magnesium sulphate to patients, administration of anti-shock garment to patients, and their knowledge of the technologies for maternal mortality reduction.

There was a significant association between the training in the use of misoprostol (p = 0.020), training in the use of magnesium sulphate (p = 0.001) and knowledge of the technologies for maternal mortality reduction with p < 0.05. This reveals that most of the people that have been trained still have a poor knowledge of the technologies, hence the need for more trainings and refresher trainings to ensure that the health care providers actually know how to use these technologies.

This study revealed that there was no significant association between training in the use of anti-shock garmentand their knowledge of the technologies for maternal mortality reduction. This may be attributed to findings from this study that reported that only about half of the respondents have ever used NASG and 88.4% of the respondents that did not use NASG said it was due to the fact that none of their patients needed it. These reasons may actually be due to the fact that the respondents need to acquire more expertise in the art of using NASG and thus save more lives by reducing maternal deaths in the state.

Of the 57 flagship PHCs reviewed in this study, 27 (47.4%) have magnesium sulphate in their facility, 42 (73.7%); have misoprotol in their facility and 52 (91.2%) have anti-shock garments in their facilities. Less than half of the PHCs sometimes run out of stock of misoprostol and magnesium sulphate in their facilities. In contrast, the Indian study mentioned earlier reported that magnesium sulphate was available in 18% of PHCs, 48% of higher public facilities and 70% of private facilities [15]. The low rate of availability of magnesium sulphate in the PHCs may be due to the fact that they rarely have cases of eclampsia and because they are quick to refer patients that are likely to have eclampsia. Another study that was done among 102 Specialist obstetricians and gynaecologists in Abuja, Nigeria in 2006 revealed that misoprostol was widely available in the environment of 82% of the respondents, whilst 14% admitted that it was either seldom or not available. Despite the popularity of the drug in Nigeria only about 53% of respondents were aware that misoprostol has been registered for use in the management of post-partum haemorrhage [17].

## Conclusion

Respondents had good knowledge of maternal mortality and its major causes and poor knowledge of the technologies for maternal mortality reduction, despite the trainings attended. Of the three the technologies considered, misoprostol was the most used. Refresher courses should be organized periodically for all the trained nurses and midwives.

## Recommendations

Trainings on the technologies should be included in the school curriculum of the nurses and midwives. Training courses on the = technologies for maternal mortality reduction should be mandatory for all employed nurses and midwives. Refresher courses should also be organized periodically for all the trained nurses and midwives.

## Supporting information

S1 File Questionnaire. (DOCX)

## Abbreviations

WHO: World Health Organization
MDG: Millennium Development Goals
PHC: Primary Health Care
PHCs: Primary Health Centres
AMTSL: Active Management of Third Stage of Labour
CHEW: Community Health Extension Worker
CHO: Community Health Officer
EmOC: Emergency Obstetric Care
HREC: Human Research Ethics Committee
LGA: Local Government Area
MAP: Mean Arterial Pressure
MgSO_4_: Magnesium Sulphate
MMR: Maternal Mortality Ratio
NASG: Non-Pneumatic Anti-Shock Garment
PHCB: Primary Health Care Board
SBA: Skilled Birth Attendant
TBA: Traditional Birth Attendant
