# Supplementary material for: Misoprostol, Magnesium Sulphate and Anti-shock garment: A knowledge, availability and utilization study at the Primary Health Care Level in Western Nigeria
Source: PLoS One. 2019 Mar 21;14(3):e0213491. doi: 10.1371/journal.pone.0213491 (PMC6460555; doi:10.1371/journal.pone.0213491)
Supplement: S1 File — (DOCX) [file pone.0213491.s001.docx]

**Supporting information**

**S1 File: Questionnaire**

**SECTION A: DEMOGRAPHIC DATA**

1. Age (in years): ___________
2. Gender: a. Male [ ] b. Female [ ]
3. Marital status:
4. Never married [ ]
5. Married / Co-habiting [ ]
6. Divorced / Separated / widowed [ ]
7. Tribe: a. Yoruba [ ] b. Hausa [ ] c. Igbo [ ] d. Others (please specify)________________
8. Religion: a. Christianity [ ] b. Islam [ ] c. Traditional religion [ ] Others________________
9. What is your qualification?
10. Nurse [ ]
11. Midwife [ ]
12. Community Health Officer/Midwife [ ]
13. Nurse/Midwife [ ]
14. Years of experience as an health care provider___________

**SECTION B: KNOWLEDGE**

1. Have you heard about the use of the following?
2. Misoprostol
3. Magnesium Sulphate
4. Anti-shock garment
5. If you have heard about any of them, what is/are your source(s) of information? (Tick as many as apply)

|  | Books | TV | Internet | Self-sponsored training | employer sponsored training | Other stakehold-ers’ sponsored training | On-the-job training |
| --- | --- | --- | --- | --- | --- | --- | --- |
| 1. Misoprostol |  |  |  |  |  |  |  |
| 1. Magnesium Sulphate |  |  |  |  |  |  |  |
| 1. Anti-shock garment |  |  |  |  |  |  |  |

1. Have you been trained in the use of the following;
2. Misoprostol
3. Magnesium Sulphate
4. Anti-shock garment
5. The following are the major causes of maternal mortality in developing countries including Nigeria.
6. Postpartum haemorrhage
7. Pre-eclampsia and eclampsia
8. Unsafe abortion
9. Sepsis
10. Obstructed labour
11. Others___________________________________
12. Don’t know
13. What is postpartum haemorrhage?
    1. Blood loss less than 200ml
    2. Blood loss less than 500ml
    3. Blood loss less than 400ml
    4. Blood loss greater than or equal to 500ml
    5. Others_____________________________________
    6. Don’t know
14. What will you assess for in a woman with postpartum haemorrhage?
    1. Vital signs (BP, pallor, temp,...)
    2. Uterine tone
    3. Haemoglobin level
    4. Others___________________________________
    5. Don’t know
15. The risk factors for postpartum haemorrhage include;
16. Mismanagement of third stage of labor
17. Prolonged labor
18. Augmented labor
19. Pre-eclampsia or eclampsia
20. Postpartum haemorrhage in previous delivery
21. Multiple gestations
22. Multiparity
23. Pregnancy induced hypertension
24. Abruptio placenta
25. Instrumental delivery
26. Placenta praevia
27. Others___________________________________
28. Don’t know
29. Common causes of postpartum haemorrhage include;
30. Uterine atony
31. Retained placenta
32. Vaginal or cervical tears
33. Coagulopathy/clotting failure
34. Others______________________________________
35. Don’t know
36. Which uterotonic(s) are used in the management of PPH?

a. Oxytocin b.Misoprostol c. Ergometrine d. Others__________________

e. Don’t know

1. Misoprostol can be used in the management of which of the following condition(s)?
2. PPH
3. Abortion
4. Induction of labour
5. Others_______________________________________
6. Don’t know
7. The required dose of misoprostol in the management of PPH is?
8. 800 μg rectal
9. 600 μg oral
10. 800 μg sublingual
11. 600 μg sublingual
12. 400 μg oral
13. Others_________________________________
14. Don’t know
15. What are the side effects of misoprostol?
16. Diarrhea
17. Pyrexia
18. Chills
19. Abdominal cramps
20. Triggering of asthma
21. Vomiting
22. Others_______________________________________
23. Don’t know
24. What is anti-shock garment used for?
25. To manage obstructed labour
26. As a first aid device to reverse hypovolaemic shock
27. To manage septic shock
28. Others_______________________________________
29. Don’t know
30. When does a patient need anti-shock garment?
    1. Blood loss less than 500ml
    2. Blood loss less than 200ml
    3. Blood loss greater than 500ml
    4. Blood loss less than 400ml
    5. Others_______________________________________
    6. Don’t know
31. Preeclampsia can be defined as which of the following;
32. Blood losx > 200ml
33. Syndrome of low blood pressure, absence of protein in the urine and swelling of the feet, legs and hands
34. Syndrome of high blood pressure, protenuria,swelling of the feet, legs, and hands.
35. Occurence of febrile convulsions with normal blood pressure
36. Others______________________________________
37. Don’t know
38. Eclampsia can be defined as which of the following;
39. Syndrome of high blood pressure, protenuria, swelling of the feet, legs, and hands.
40. Occurrence of one or more convulsions in association with the syndrome of pre-eclampsia
41. Blood loss of >3,000ml
42. Syndrome of low blood pressure, absence of protein in the urine and swelling of the feet, legs and hands
43. Others______________________________________
44. Don’t know
45. The following are the signs and symptoms of Eclampsia:
46. Headache
47. Hypereflexia
48. Blurred vision
49. Oliguria
50. Epigastric pain
51. Pulmonary oedema.
52. Convulsion
53. Nausea and vomiting
54. Others_______________________________________
55. Don’t know
56. What will you asses in a woman suspected of having preeclampsia/eclampsia?
57. Blood pressure
58. Level of consciousness
59. Fetal heart rate
60. Rapidly developing oedema
61. Urine for protein
62. Others_____________________________________
63. Don’t know
64. The following can be used in the management of preeclampsia and eclampsia.
65. Magnesium Sulphate
66. Anti-hypertensives
67. Oxygen
68. Others_____________________________________
69. Don’t know
70. What is the required dose of IM magnesium sulphate
71. 5g IV loading dose followed immediately by 10g IM, then 4g IM 4hrly in alternating buttocks.
72. 4g IV loading dose given slowly over 5 – 10 minutes followed immediately by 10g IM, then 5g IM 4hrly in alternating buttocks.
73. 6g IV loading dose followed immediately by 15g IM, then 4g IM 4hrly in alternating buttocks.
74. 4g IV loading dose given as a push followed immediately by 10g IM, then 5g IM 4hrly in alternating buttocks.
75. Others___________________________________
76. Don’t know
77. What is the required dose of IV magnesium sulphate
78. IV regimen of 3 g loading dose, followed by a maintenance infusion of 1 to 5g/h by controlled infusion pump.
79. IV regimen of 5 g loading dose over 1 minute, followed by a maintenance infusion of 1 to 4g/h by controlled infusion pump.
80. IV regimen of 4 g loading dose slowly over 5 - 10 minutes, followed by a maintenance infusion of 1 to 2g/h by controlled infusion pump.
81. IV regimen of 5g dose given as a push, followed by a maintenance infusion of 4 to 6g/h by controlled infusion pump.
82. Others____________________________________
83. Don’t know
84. Clinical conditions in which magnesium sulphate can be used are?
85. Prevention and treatment of seizures in women with preeclampsia or eclampsia.
86. Fetal neuroprotection before anticipated early preterm (less than 32 weeks of gestation) delivery.
87. Short-term prolongation of pregnancy (up to 48 hours) to allow for the administration of antenatal corticosteroids in pregnant women between 24 - 34 weeks of gestation who are at risk of preterm delivery within 7 days.
88. Others_________________________________________
89. Don’t know
90. Side effects of magnesium sulphate is/are?
91. Respiratory depression
92. Decreased urinary output
93. Depression of deep tendon reflexes
94. Abdominal cramps
95. Triggering of asthma
96. Vomiting
97. Others_________________________________________
98. Don’t know

**SECTION C: UTILIZATION**

1. Have you ever managed a patient with PPH?

a. Yes b. No

1. Have you ever administered misoprostol to any patient?
   1. Yes b. No If no, go to question 37.
2. If yes, how often?
   1. Rarely
   2. Sometimes
   3. Often
   4. Always
3. When was the last time you used misoprotol?
4. Less than 1 month ago
5. Between 1 month – <3 months ago
6. Between 3 months – 6 months ago
7. More than 6 months ago
8. What was the clinical condition of the last patient you gave misoprostol?
9. Obstructed labour
10. Post-partum haemorrhage
11. Eclampsia
12. Hypovolaemic shock
13. Others_______________________________
14. What was the outcome of that patient?

a. Recovery b. Referral c. Death

1. If no to question 32, Why not?

a. Didn’t know it was available b. No patient needed it c. Don’t know how to use it d. Others_________________________

1. Have you ever administered magnesium sulphate to any patient?
2. Yes b. No If no go to question 43.
3. If yes, how many often?
   1. Rarely
   2. Sometimes
   3. Often
   4. Always
4. When was the last time you used magnesium sulphate?
   1. Less than 1 month ago
   2. Between 1 month – <3 months ago
   3. Between 3 months – 6 months ago
   4. More than 6 months ago
5. What was the clinical condition of the last patient you gave magnesium sulphate?
   1. Obstructed labour b. Post-partum haemorrhage c. Eclampsia

d. Preeclampsia e. Others_______________________

1. What was the outcome of that patient?
   1. Recovery b. Referral c. Death
2. If No to question 38, why not?
3. Didn’t know it was available b. No patient needed it

c. Don’t know how to use it d. Others________________________

1. Have you ever administered anti-shock garment to any patient?
2. Yes b. No If no go to question 49.
3. If yes, how many often? ___________
   1. Rarely
   2. Sometimes
   3. Often
   4. Always
4. When was the last time you used anti-shock garment?
   1. Less than 1 month ago
   2. Between 1 month – <3 months ago
   3. Between 3 months – 6 months ago
   4. More than 6 months ago
5. What was the clinical condition of the last patient you used anti-shock garment for?
   1. Obstructed labour b. Post-partum haemorrhage c. Eclampsia

d. Hypovolaemic shock e. Others_______________________

1. What was the outcome of that patient?
   1. Recovery b. Referral c. Death
2. If No to question 44, why not?
3. Didn’t know it was available b. No patient needed it

c. Don’t know how to use it d. Others ____________________

**CHECKLIST FOR AVAILABILITY**

1. Do you have magnesium sulphate in your facility?

Yes No

2. If Yes, How many vials do you have?

1-5 6-10 11-15 16-20 >20

3. Do you have misoprostol in your facility?

Yes No

4 If yes, how many cartons do you have?

1 2 3 4 >5

5. Do you have anti-shock garments in your facility?

Yes No

6. If yes, how many do you have? ___________

7. Do you have blood pressure measuring apparatus in your facility?

Yes No

8. Do you have stetoscope in your facility?

Yes No

9. Do you have oxygen cylinder with regulator and mask in your facility?

Yes No

10. Do you have fetoscope in your facility?

Yes No

12. Does the facility have access to transportation during emergencies?

Yes No

13. What are the number of deliveries in the preceeding month?__________________

Normal delivery (SVD) Assisted Delivery

14. Do you sometimes run out of stock of misoprostol?

Yes No

15. Do you sometimes run out of stock of magnesium sulphate?

Yes No
